# Harnessing mRNA to unleash endolysins: A new frontier in antibacterial therapy

**DOI:** 10.1016/j.omtn.2024.102249

**Published:** 2024-06-26

**Authors:** Daniel C. Nelson, Urmil M. Dave, Norberto Gonzalez-Juarbe

**Affiliations:** 1 Institute for Bioscience and Biotechnology Research, University of Maryland, Rockville, MD 20850, USA; 2 Department of Veterinary Medicine, University of Maryland, College Park, MD 20742, USA; 3 Department of Cell Biology and Molecular Genetics, University of Maryland, College Park, MD 20742, USA

## Main text

The study by Jansson et al.^1^ describes a unique approach to combat bacterial infections by using mRNA technology to express endolysins in human cell lines. Endolysins are bacteriophage-derived cell wall hydrolases that function to degrade the bacterial peptidoglycan and, as such, are gaining attention for their potential use as bacteriolytic agents against sensitive organisms. This strategy aims to provide a mechanism to target endolysins directly to the foci of infection for localized production. By designing mRNA constructs, the researchers successfully induced the expression of the pneumococcus-specific endolysin Cpl-1 in three different human cell lines and demonstrated that the heterologous produced enzyme retained antibacterial activity against *Streptococcus pneumoniae*. The constructs also included a human lysozyme signal peptide in the Cpl-1 sequence, which directed the enzyme to the secretory pathway for extracellular secretion. Furthermore, the incorporation of a point mutation to prevent N-linked glycosylation resulted in a modified variant, hlySP-sCpl-1N215D, which showed improved bacteriolytic activity against pneumococci. This work demonstrates the potential of mRNA-based endolysin therapy for pneumococcal disease and suggests that the platform may be applicable to other bacterial pathogens.

The advent of mRNA technology has revolutionized various medical fields, most notably in vaccine development, but also including cancer treatment and enzyme replacement therapies for diseases such as hemophilia and Fabry disease.^2^ The success of mRNA vaccines against COVID-19 underscores the potential of this technology to provide rapid and effective responses to infectious diseases.^3^ The study by Jansson et al.^1^ leverages this technology to address the emergence of antibiotic-resistant organisms, which poses a significant threat to public health. Rather than using synthetic mRNA to encode an antigen, which is the hallmark of vaccine approaches, the investigators encoded endolysins within the mRNA. Once expressed, endolysins rapidly hydrolyze the peptidoglycan of susceptible bacteria, resulting in the osmotic lysis of the organism. Their bacteriolytic action renders endolysins invulnerable to common resistance mechanisms associated with traditional antibiotics, such as penicillin-binding proteins, efflux pumps, and alterations in metabolic pathways.^4^ Additionally, due to unique cell wall binding domains found in many endolysins, these enzymes are often species or genus specific, making them narrow-spectrum antimicrobial therapeutics that have little impact on the beneficial microbiome. At least four endolysins have reached human clinical trials, and in 2020, the US Food and Drug Administration granted the “Breakthrough Therapy” designation for an endolysin in a phase III trial targeting methicillin-resistant *Staphylococcus aureus*.^5^

**Figure 1:**
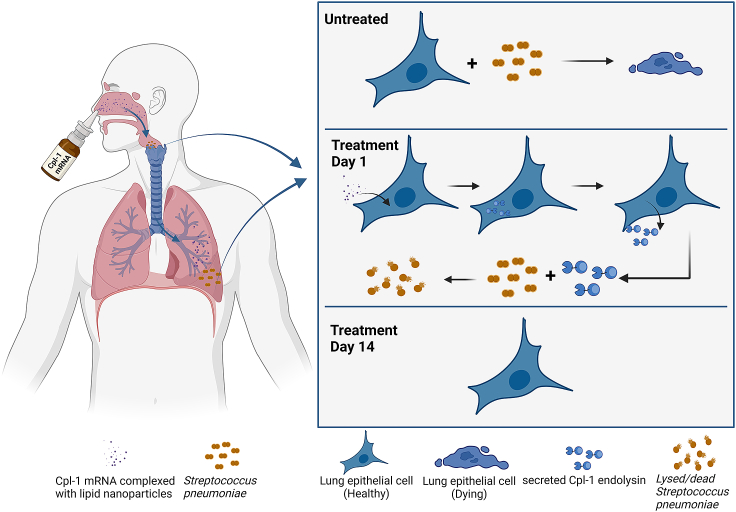
While untreated cells can be damaged by *S. pneumoniae*, the Cpl-1 endolysin will start to be expressed and secreted as soon as 12 h post mRNA treatment By day 14 post-treatment, cells would have recovered from the infection, and the mRNA would have degraded, thereby circumventing any long-term concerns associated with the injection of foreign nucleic acids.

mRNA technology provides a delivery strategy by enabling cells within target organs or tissues to produce endolysins endogenously. This approach has several advantages: it bypasses the need for repeated dosing over a several-day period, it can enhance bioavailability of proteins that may be rapidly degraded or inactivated by local environmental factors, and it can allow for targeted delivery.^6^ For instance, mRNA delivery systems can be administered directly to the tissues where bacterial colonization or infection is prevalent. As suggested by the current study, an aerosolized delivery of mRNA could be used to transfect lung epithelial cells, which, in turn, would secrete the manufactured Cpl-1 endolysin, resulting in localized anti-pneumococcal activity (Figure 1).

The investigators methodically optimized their mRNA constructs and evaluated the resulting expression patterns. Notably, the 5′ and 3′ untranslated regions (UTRs) were stabilized with human alpha-globin UTRs, a 5′ cap-1 structure (i.e., methylation of the first base) was included, and a poly(A) tail of 120 bases was added to the 3′ end. In addition, uridine was replaced with N^1^-methylpseudouridine to reduce immunogenicity of the mRNA construct itself, as described previously.^7^ Last, seven different signal peptides were evaluated. While the human lysozyme signal peptide was ultimately selected as the best, all seven were able to secrete Cpl-1, with the signal peptides for human immunoglobulin G heavy chain and human azurocidin being nearly as effective as the lysozyme signal peptide. mRNA dosing studies and transfection time were also investigated across several cell lines. These foundational experiments not only set a precedent for mRNA-based expression of endolysins but will act as an important guide for the endolysin community as it embraces the potential of this technology.

Whereas the site of administration for an mRNA-based vaccine does not have a significant impact on presentation of the expressed antigen to the immune system, mRNA-based therapeutic proteins, including endolysins, must be expressed at the site of disease or infection. Toward this end, the literature is replete with endolysins that have been characterized *in vitro* and *in vivo* against pathogens that colonize or infect discrete niches in the body. For example, staphylococcal or streptococcal endolysins delivered via mRNA to the skin could help alleviate atopic dermatitis or eczema. Oral mucosal delivery of endolysin mRNA could be used to address pneumococcal colonization of the nasopharynx or streptococcal pharyngitis. Delivery to the lungs via a nebulizer could target pneumococcal pneumonia, as envisioned in the current study (Figure 1), or *Pseudomonas aeruginosa* in cystic fibrosis patients. Delivery to the vaginal epithelium could be beneficial for symptoms of bacterial vaginosis caused by *Gardnerella* species or to decolonize group B *Streptococcus* perinatally. Intravesical delivery of endolysin mRNA to the bladder epithelia could treat pathogens implicated in catheter-associated urinary tract infection. Finally, application of endolysins via mRNA directly to a surgical wound or prosthetic joints could lessen postsurgical infection or osteomyelitis.

While the present study demonstrates the feasibility of mRNA-based endolysin therapy, several important considerations remain unaddressed. First and foremost, the *in vitro* nature of the experiments necessitates further *in vivo* studies to evaluate the efficacy and safety of this approach. Additionally, the stability and bioavailability of the mRNA constructs in different body environments or specific tissues will be crucial for the success of this therapy. Another limitation of localized lytic therapy pertains to pathogens with soluble virulence determinants that are released upon lysis. In the pneumococcal pneumonia example, simultaneous lysis of a high titer of bacteria could release a bolus of pneumolysin (a cytotoxic pore-forming toxin), which can lead to exacerbated inflammation, lysis of airway cells, and death.^8^ Thus, controlled effector expression may serve as a potential research strategy to develop new respiratory and mucosal therapeutics.

In conclusion, the application of mRNA technology to endolysin therapy offers a promising new avenue for combating antibiotic-resistant bacterial infections. The study provides a proof of concept for this approach, demonstrating significant antibacterial activity against *S*. *pneumoniae*. As research progresses, this technology could be adapted to target a wide range of bacterial pathogens, providing a versatile and effective tool in the fight against infectious diseases.
